# State diagrams of the heart – a new approach to describing cardiac mechanics

**DOI:** 10.1186/1476-7120-7-22

**Published:** 2009-05-27

**Authors:** Matilda Larsson, Anna Bjällmark, Jonas Johnson, Reidar Winter, Lars-Åke Brodin, Stig Lundbäck

**Affiliations:** 1 School for Technology and Health, Royal Institute of Technology, Alfred Nobels Allé 10, SE-141 52 Huddinge, Sweden; 2 Department of Clinical Physiology, Karolinska University Hospital, SE-141 86 Stockholm, Sweden; 3 GrippingHeart AB, KTH Business Lab, Teknikringen 26, SE-114 28 Stockholm, Sweden

## Abstract

**Background:**

Cardiac time intervals have been described as a measure of cardiac performance, where prolongation, shortening and delay of the different time intervals have been evaluated as markers of cardiac dysfunction. A relatively recently developed method with improved ability to measure cardiac events is Tissue Doppler Imaging (TDI), allowing accurate measurement of myocardial movements.

**Methods:**

We propose the state diagram of the heart as a new visualization tool for cardiac time intervals, presenting comparative, normalized data of systolic and diastolic performance, providing a more complete overview of cardiac function. This study aimed to test the feasibility of the state diagram method by presenting examples demonstrating its potential use in the clinical setting and by performing a clinical study, which included a comparison of the state diagram method with established echocardiography methods (E/E' ratio, LVEF and WMSI). The population in the clinical study consisted of seven patients with non ST-elevation myocardial infarction (NSTEMI) and seven control subjects, individually matched according to age and gender. The state diagram of the heart was generated from TDI curves from seven positions in the myocardium, visualizing the inter- and intraventricular function of the heart by displaying the cardiac phases.

**Results:**

The clinical examples demonstrated that the state diagram allows for an intuitive visualization of pathological patterns as ischemia and dyssynchrony. Further, significant differences in percentage duration between the control group and the NSTEMI group were found in eight of the totally twenty phases (10 phases for each ventricle), e.g. in the transition phases (Pre-Ejection and Post-Ejection). These phases were significantly longer (> 2.18%) for the NSTEMI group than for the control group (p < 0.05). No significant differences between the groups were found for the established echocardiography methods.

**Conclusion:**

The test results clearly indicate that the state diagram has potential to be an efficient tool for visualization of cardiac dysfunction and for detection of NSTEMI.

## Introduction

Cardiac time intervals have been described as a measure of cardiac performance and as a tool to help understand and illustrate the mechanics of the heart. Prolongation, shortening and delay of the different time intervals have been evaluated as markers of cardiac dysfunction [1,2]. Methods to measure the time intervals in the cardiac cycle include electrocardiography [3], phonocardiography [4], apexcardiography [5], and echocardiography [6]. A relatively recently developed method with improved ability to measure cardiac events is Tissue Doppler Imaging (TDI), which allows for accurate measurement of cardiac phases generated by movement of the myocardium [7]. Different approaches of dividing the cardiac cycle into phases have been reported in the literature [8-11]. In a TDI-velocity curve, the most common way is to divide systole into two phases and diastole into four phases. The systolic phases are the isovolumic contraction time (IVCT) and the ejection phase, whereas the diastolic phases are the isovolumic relaxation time (IVRT), the early diastolic filling, the diastase and the late diastolic filling (atrial contraction) [9].

We propose the state diagram of the heart as a new visualization tool for cardiac time intervals, presenting comparative, normalized data of systolic and diastolic performance, providing a more complete overview of cardiac function [12]. This tool is based on a long period of research concerning the heart working according to a novel pump principle referred to as the Dynamic Displacement Pump (DDP) [13-15]. With this pump principle and new definitions of the cardiac phases it is possible to visualize the hearts mechanical function as state diagrams. According to the DDP-principle, the heart pumps with the back and forth going movements of a piston-like unit, referred to as the dome-shaped AV-piston, which is divided into a left and a right AV-piston by the inter-ventricular septum (IVS). The common AV-piston consists of the ring of annulus fibrosis with its four valves and the dome-shaped muscular tissue of the ventricles in connection to the ring of annulus fibrosis. When the AV-pistons are drawn towards the apex of the heart and the blood in the ventricles is forced into the pulmonary and systemic circulation, they will at the same time by suction create an inflow to the atria. The dome-shaped parts of the AV-pistons that border to the surroundings of the heart and the areas that are generated by the orifices of the outflow vessels will by motions of the AV-pistons generate extracardiac volume changes. These volumes are partly responsible for the hydraulic return of the pistons and the more or less continuous inflow to the heart [13].

State diagrams are used to describe the behaviour of systems, often applicable in technical systems in order to explain and simplify the way the system operates. This study is the first attempt, in a comprehensive work, to present the function of the right and left side of the heart using state diagrams. The work has, in particular, focused on rational ways to find the mechanical criteria for how the heart goes from one state to the next, which defines the regional and global function of the heart. The duration of the different cardiac states, heart phases, are presented in the state diagram. The primary aim of the study was to test the feasibility of the state diagram method by performing a clinical study, which included a comparison of the state diagram method with established echocardiography methods, and by presenting clinical examples demonstrating its potential use in the clinical setting.

## Methods

### Image acquisition

The state diagrams were generated from myocardial velocity curves recorded by TDI. Image acquisition was performed in the left lateral decubitus position using a Vingmed System Vivid 7 (GE Vingmed Ultrasound, Horten, Norway) with a standard 2D transducer (M3S) or a 3D matrix transducer (3V) for triplane imaging. Images were obtained in three imaging planes: apical long-axis (APLAX), apical four-chamber (4CH) and apical two-chamber (2CH) and were imported to EchoPAC Dimension (GE Vingmed Ultrasound, Horten, Norway). In the two-dimensional gray scale image with superimposed colour Doppler, a circular region of interest (ROI) with a diameter of 4 mm was positioned in the basal segment of the antero-septal, anterior, antero-lateral, infero-lateral, inferior, infero-septal (left ventricle) and medial wall (right ventricle, 4CH view). The mean velocity curve for each ROI, in total seven, was stored digitally in a format compatible with Matlab R2006b, where the software was developed. The study has been carried out in accordance to the declaration of Helsinki and ethical approval was obtained from the local ethics committee. All participants gave informed consent.

### Definition of cardiac phases

**Figure 1:**
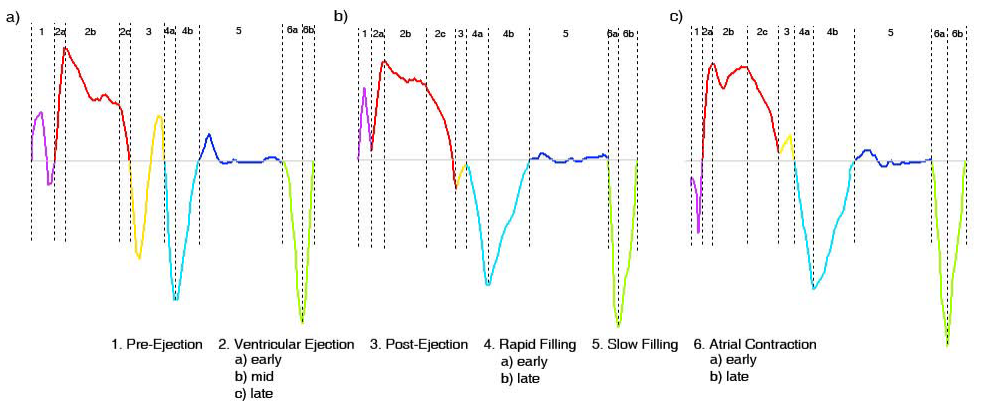
**Definition of cardiac phases**. Typical tissue Doppler imaging echocardiography-derived velocity profiles. a) Definition of cardiac phases in a velocity curve containing typical biphasic pattern with a negative and a positive velocity spike during Pre-Ejection and Post-Ejection. b and c) Definition of cardiac phases in a velocity curve containing only one zero-crossing during Pre-Ejection and Post-Ejection.

The generation of a state diagram was initiated with a detection of triggering points in each tissue velocity curve. The cardiac cycle was divided into six main phases in the tissue velocity curve: Pre-Ejection, Ventricular Ejection, Post-Ejection, Rapid Filling, Slow Filling and Atrial Contraction. Ventricular Ejection was subdivided into an early, a mid and a late phase whereas the Atrial Contraction and the Rapid Filling were subdivided into two phases: a early phase and a late phase. Figure 1 illustrates how the criteria for start- and endpoints of all the phases were applied, dependent on different appearances of the velocity curve.

Pre-Ejection was defined as the time interval between the first zero-crossing in the tissue velocity curve after the R-wave in the ECG-curve and the zero-crossing after the typical biphasic pattern with a negative and a positive velocity spike, which are involved in the closure of the inlet valves. This occurs at the ascending limb of the tissue velocity curve at the beginning of Ventricular Ejection, see Figure 1a. In cases with only one ascending zero-crossing during Pre-Ejection, the start- and endpoints were defined as illustrated in Figure 1b and 1c.

Ventricular Ejection was defined as the time interval between the end of Pre-Ejection and of the start of the Post-Ejection phase. Early Ventricular Ejection was defined as the first acceleration phase lasting until the largest derivative change in the ascending limb of the tissue velocity curve. Late Ventricular Ejection was defined as starting with the largest derivative change in the descending limb of the tissue velocity curve. The time between early and late Ventricular Ejection was called mid Ventricular Ejection.

Post Ejection was defined as the time interval between the zero-crossing of the descending limb of the systolic tissue velocity curve and the zero-crossing after the biphasic movements with a negative and a positive velocity spike, which are involved in the closing procedure of the outlet valves, see Figure 1a. In cases with only one descending zero-crossing during the Post-Ejection phase, the start- and endpoints were defined as illustrated in Figure 1b and 1c.

Rapid Filling was defined as the time between the endpoint of Post-Ejection and the time point when the velocity derivative shifts from a positive value to a value close to zero. The phase was divided into an early and a late part by the minimum velocity value during this phase.

Slow Filling was defined as the time from the end of Rapid Filling until the start of Atrial Contraction.

Atrial Contraction was defined as starting with the largest decline in the tissue velocity curve occurring in connection to the P-wave in the ECG. The end point equalled the start of the Pre-Ejection phase for the next cardiac cycle. The Atrial Contraction was divided into an early and a late phase with the minimum velocity value as the dividing event.

### Visual interpretation of the state diagram

**Figure 2:**
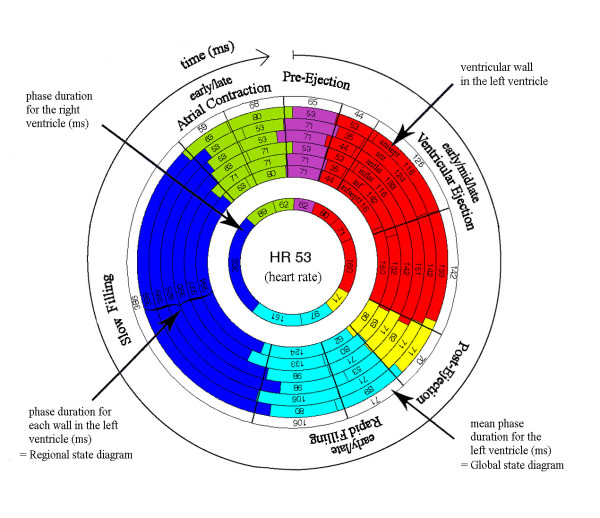
**Visual interpretation of the state diagram**. One heart beat corresponds to all 360° in the state diagram, starting with Pre-Ejection and ending with Atrial Contraction. The state diagram contains regional information from seven positions in the heart and global information represented by the mean time intervals. The outermost circular segment in the state diagram corresponds to the antero-septal wall, followed by the anterior, antero-lateral, infero-lateral, inferior, infero-septum (LV) and medial wall (RV). The global function of the left ventricle was an average of six measuring points, while the only measuring point in the right ventricle represented regional function.

The outermost circular segment in the state diagram corresponds to the antero-septal wall, followed by the anterior, antero-lateral, infero-lateral, inferior, infero-septum and medial wall, see Figure 2. One heart beat corresponds to all 360° in the circle, starting with Pre-Ejection and ending with Atrial Contraction. Every phase is expressed by a colour and presented with its duration. The purpose of the colour coding is to visualize the duration of each phase, the transition from one phase to the next and, in particular, the relationship between different phases in the cardiac cycle. The purple colour was assigned to represent Pre-Ejection, the red colour Ventricular Ejection, the yellow colour Post-Ejection, the light blue Rapid Filling, the dark blue Slow Filling and the green colour Atrial Contraction. The state diagram contains regional information from seven positions in the heart and global information represented by the mean time intervals. The global function of the left ventricle was an average of six measuring points, while the only measuring point in the right ventricle represented regional function. The regional function will henceforth be referred to as "the regional state diagram" and the global function as "the global state diagram". If there was a time delay between the walls, i.e. a difference in the start of Pre-Ejection, the phases were shifted, clockwise or counterclockwise in the state diagram, relative to the antero-septal wall. The calculation of the time shift between the walls was derived from the time between the R-wave in the ECG-curve and the start of Pre-Ejection.

### Demonstration of clinical examples

**Figure 3:**
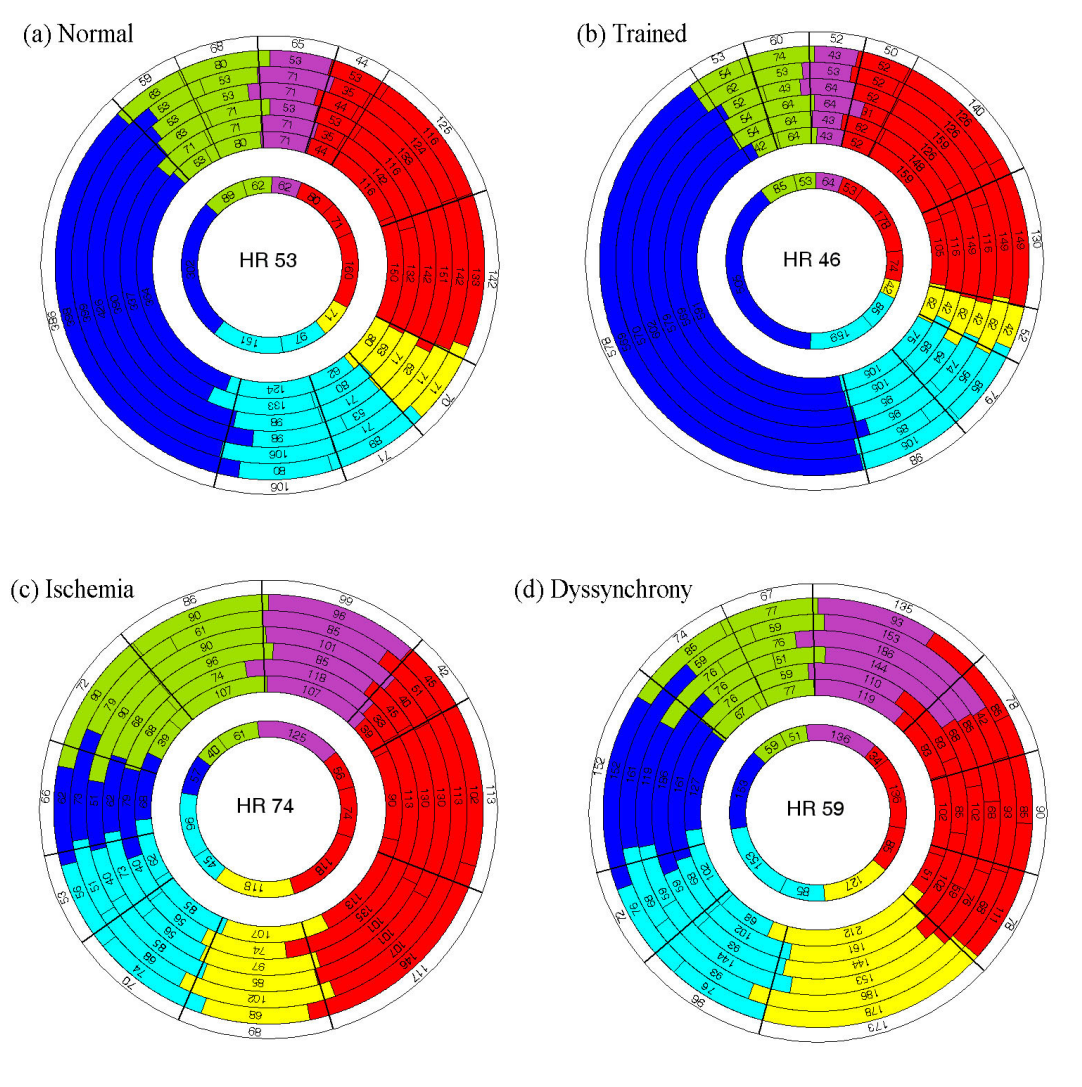
**State diagrams in case examples**. a) State diagram in a healthy subject b) State diagram in an athlete. c) State diagram in a subject with ischemia. d) State diagram in a subject with cardiac dyssynchrony.

State diagrams were generated at rest in four typical cases, the healthy subject, the athlete, the ischemic subject and the dyssynchronic subject. Figure 3a illustrates a state diagram in a healthy subject with no known heart disease (female, 24 years old, 100 frames/s, 3D transducer). Figure 3b displays a state diagram in an athlete, a professional basketball player (female, 26 years old, 94.8 frames/s, 3D transducer). Figure 3c displays a state diagram in an ischemic subject with acute non ST-elevation myocardial infarction (NSTEMI) due to mid LAD-stenosis (male, 74 years old, 154 frames/s, 2D transducer) and Figure 3d gives an example of a state diagram visualizing a patient with intra-ventricular dyssynchrony in the antero-lateral wall (female, 56 years old, 154.2 frames/s, 3D transducer).

### Clinical study

A sample of seven patients with NSTEMI was randomly selected from the echo archive at Karolinska University Hospital in Huddinge, Sweden. The control group was individually matched according to age and gender, with no symptoms of ischemic heart disease at the time for the investigation. Each group consisted of 6 men and 1 woman. The mean age was 58.2 (SD 7.6) in the diseased group and 58.7 (SD 8.0) in the control group and the mean heart rate was 61.0 (SD 5.7) and 62.4 (SD 6.6), respectively. The characteristics of the studied subjects are described in Table 1.

**Table 1:** Patient characteristic

Subject	Age	Gender	HR	Clinical features	Medications
NSTEMI	49	male	65	Hypertension, hyperlipidemia	ASA, Clopidogrel, betablocker, statin, calcium antagonist
NSTEMI	53	male	70	Smoker, hypertension, hyperlipidemia	ASA, ACE1, Clopidogrel, betablocker, statin, calcium antagonist, diuretics
NSTEMI	55	female	63	Smoker, psoriasis	ASA, Clopidogrel, betablocker, statin
NSTEMI	56	male	61	Smoker	ASA, Clopidogrel, betablocker, statin
NSTEMI	60	male	59	Renal failure	ASA, Clopidogrel, statin,
NSTEMI	63	male	56	Smoker, hypertension	ASA, Clopidogrel, betablocker, statin, ACE-inhibitor,
NSTEMI	72	male	53	Diabetes II	ASA, Clopidogrel, betablocker, statin, ACE-inhibitor, Metforminen, Glibenclamide
					
Control	49	male	60	Ex-smoker, hypertension, diabetes II	ASA, ACE1, betablocker, calcium antagonist, metformine
Control	52	male	59	Myeloma	Talidomide, steroids, ASA
Control	54	female	57	Diabetes II, Bypass 2004	Statin, betablocker, calcium antagonist, metformine
Control	59	male	71	Hypertension, liver cirrosis	-
Control	62	male	66	Ex-smoker, hypothyreosis	Levaxine
Control	62	male	70	Atrial fibrillation	Betablocker
Control	73	male	54	-	-

Patient characteristic for the NSTEMI group and their matched controls.

Conventional two-dimensional echocardiography and colour TDI images were recorded for both groups. Images were obtained at rest as described above with a standard 2D transducer (M3S) with a frame rate of the TDI data varying between 100 and 150 frames/s. The flow velocity (E) and the mean tissue velocity (E') of the early diastolic inflow in the septal and lateral corner of the mitral annulus were used for calculation of the E/E' ratio. Left ventricular Ejection Fraction (LVEF) was measured with the biplane Simpson's method and wall motion score index (WMSI) was performed by an experienced stress echo reader. State diagrams for each subject were generated with the software developed in Matlab, see above for more details.

### Statistical Analysis

All statistical analysis was performed using standard statistical software (SPSS version 16.0). For the individually matched couples, a paired t-test was performed to test the difference in percentage duration of each phase in the global state diagram (control-NSTEMI). A total of twenty phases were tested, ten in the left ventricle and ten in the right ventricle. A paired t-test was also used to test the difference in E/E' ratio, LVEF and WMSI between the groups.

## Results

For the totally twenty phases in the state diagram, significant differences in percentage duration between the control group and the NSTEMI group were found in eight. When the values were positive, the percentage duration was longer for the control group than for the NSTEMI group. In the late Atrial Contraction, significant differences were found for both ventricles, 2.27% SD 1.5 (p = 0.008) for the left ventricle and 2.40% SD 1.6 (p = 0.007) for the right ventricle. The difference in percentage duration of Pre-Ejection was significant in both ventricles, -3.52% SD 1.5 (p = 0.001) and -2.18% SD 2.2 (p = 0.041) for the left and the right ventricle, respectively. The difference in percentage duration for mid Ventricular Ejection in the right ventricle was 2.09% SD 2.1 (p = 0.038) and for Post-Ejection, -2.70% SD 2.5 (p = 0.029) for the left and -2.94% SD 1.5 (p = 0.002) for the right ventricle. For early Rapid Filling in the left ventricle the difference in percentage duration was 1.65% SD 1.5 (p = 0.026).

**Figure 4:**
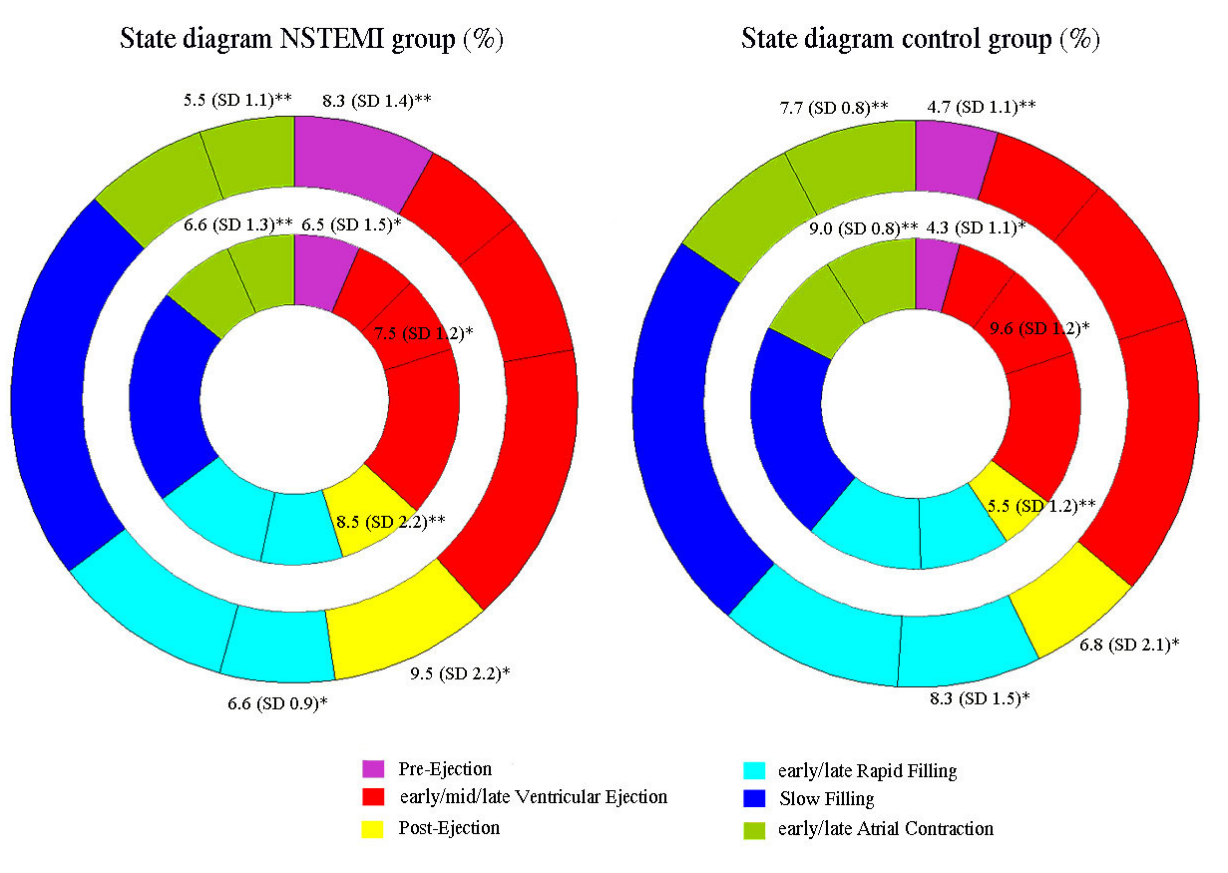
**Comparison between the NSTEMI group and the control group**. Global state diagram for the NSTEMI group and the control group. The mean percentage of duration for the phases is displayed with corresponding level of significance for each group.

The results from the statistical analysis regarding the state diagram variables are shown in Table 2. The mean percentage (%) of each phase is displayed with its standard deviation for both groups and ventricles. The level of significance is illustrated with annotations (* p < 0.05, ** p < 0.005). Figure 4 illustrates the mean state diagram for the NSTEMI-group and the control group. The mean heart rate in the study was 62.7 beats/s, which results in a mean cardiac cycle duration of 957 ms. Accordingly, 1% of the duration of one cardiac cycle corresponds to approximately 10 ms. The mean percentage of duration for the phases is displayed with corresponding level of significance in Figure 4 for each group.

**Table 2:** Comparison of mean percentage duration (%) in cardiac phases between the NSTEMI group and the control group

	Left ventricle			Right ventricle		
	NSTEMI group mean (SD)	Control group mean (SD)	Difference mean (SD)	NSTEMI group mean (SD)	Control group mean (SD)	Difference mean (SD)

Pre-Ejection	8.26 (1.4)	4.73 (1.05)	-3.52 (1.5)**	6.49 (1.5)	4.31 (1.5)	-2.18 (2.2)*
Early Ventricular Ejection	6.02 (1.4)	6.55 (1.8)	0.53 (1.5)	5.98 (1.4)	6.00 (1.9)	0.02 (2.2)
Mid Ventricular Ejection	8.12 (1.5)	9.07 (0.8)	0.95 (2.0)	7.52 (1.2)	9.61 (1.2)	2.09 (2.1)*
Late Ventricular Ejection	15.31 (1.5)	15.16 (2.4)	-0.15 (2.5)	16.78 (1.8)	15.21 (2.7)	-1.57 (4.0)
Post-Ejection	9.46 (2.2)	6.76 (2.1)	-2.70 (2.5)*	8.45 (2.2)	5.51 (1.2)	-2.94 (1.5)**
Early Rapid Filling	6.60 (0.9)	8.25 (1.5)	1.65 (1.5)*	8.08 (1.3)	8.83 (2.0)	0.75 (1.6)
Late Rapid Filling	10.49 (1.3)	10.64 (1.4)	0.15 (2.2)	11.46 (3.3)	11.52 (2.0)	0.06 (4.13)
Slow Filling	23.13 (6.0)	23.25 (4.2)	0.12 (9.1)	21.14 (4.4)	21.66 (4.3)	0.52 (8.3)
Early Atrial Contraction	7.12 (0.5)	7.85 (0.8)	0.73 (1.2)	7.46 (0.9)	8.32 (1.8)	0.86 (1.6)
Late Atrial Contraction	5.47 (1.1)	7.74 (0.8)	2.27 (1.5) **	6.63 (1.3)	9.03 (0.8)	2.40 (1.6)**

The mean percentage duration (%) of each phase is displayed with its standard deviation for both the groups and the ventricles. The right column for both ventricles contains differences in percentage distribution of the phases in the state diagram between the NSTEMI group and the control group. The level of significance is illustrated with annotations (* p < 0.05, ** p < 0.005).

Table 3 shows the results from the comparison of E/E' ratio, LVEF and WMSI between the groups. The mean, the mean difference (control-NSTEMI) and the standard deviation for each variable are displayed for the NSTEMI group and the control group. No significant differences between the groups were found in these variables.

**Table 3:** Comparison of echocardiographic variables between the NSTEMI group and the control group

	NSTEMI group mean (SD)	Control group mean (SD)	Difference mean (SD)
E/E' ratio	13.66 (4.6)	10.51 (4.4)	-3.15 (6.2)
LVEF (%)	57.57 (12.2)	59.00 (4.9)	1.43 (9.9)
WSMI	1.20 (0.3)	1.01 (0.0)	-0.19 (0.3)

The table displays the mean and the mean difference in E/E' ratio, left ventricular Ejection Fraction (LVEF) and wall motion score index (WMSI), with the standard deviation for the NSTEMI group and the control group.

## Discussion

This study was an attempt to test the clinical feasibility of the state diagram as a visualization tool for cardiac mechanics. The state diagram was designed to present the mechanics of both the left and the right side of the heart since they each have an AV-piston. The myocardium is the power source for the pumping function. It has a flexible structure with a constant volume, which means that generation and release of tension forces within the myocardium can occur with low or no external volume changes, i.e. changes of the epicardial borders [16]. The myocardium has to build up enough tension before there can be any flow out of the ventricle and must then release this tension before the filling of the ventricles can occur. The global function of the heart, represented by the global state diagram, results in flow and pressures into and out of the right and left side of the heart. The global function is dependent on many complex regional functions and external conditions. The fact that the heart has to build up and release enough tension before there can be any large flow or pressure changes partly explains why there is a time delay between different regional muscular movements and global events. The regional velocity curves can either be an active movement contributing to the global function or passive, i.e. as a result of movements occurring at other sites in the heart. In this way the velocity curves contain both events useful to generate the global state diagram from the right and left ventricles and events to create local state diagrams that depict the local functions at different sites of the two ventricles.

The phases in the state diagram have been defined in accordance with the DDP-principle in order to illustrate the mechanics of the heart, and henceforth the heart will be described as a DDP. Since the motion of the AV-pistons largely represents the mechanics of the heart, the myocardial velocities were chosen to be measured in the basal segments of the heart, near the AV-pistons. The crucial task for the heart is to preserve flow and muscle dynamics throughout the cardiac cycle and to keep a constant inflow to the heart when the AV-pistons have to be accelerated, retarded and change direction. The more or less constant inflow to the heart is achieved by suction of blood into the atria during Ventricular Ejection and continuous need of further inflow during the hydraulic return of the AV-piston during Rapid and Slow Filling.

Motion shifts of the piston associated with opening and closing of the valves are critical events that affect the performance of the heart. These events have in this study been included in the phases Pre-Ejection and Post-Ejection, names which refer to their close link to the beginning and ending of Ventricular Ejection. Pre-Ejection and Post-Ejection differ from the traditionally defined isovolumic phases by including volume redistributions between the atria, the ventricles and its outlets. During Atrial Contraction, the AV-pistons are drawn towards the inlet areas of the atria, the base of the heart, whereas during Pre-Ejection they are drawn towards the apex of the heart [17,18]. This will result in rearrangement of blood volumes from the atria to the ventricles during Atrial Contraction and in the reverse direction during Pre-Ejection. In this way the stroke-length of the AV-pistons can increase and the closure of the tricuspid and mitral valves can occur with small disturbances in the inflow to the heart.

Events connected to Pre-Ejection and Post-Ejection are considered to result from pre- and postsystolic reshaping of the muscles in the ventricles [19]. Pre-Ejection was defined starting with the beginning of the closure of the tricuspid and the mitral valves, i.e. the generation of muscle tension and blood volume redistributions. This phase ends with a powerful tension increase that initiates the opening of the pulmonic and aortic valves. Observe that the flow out of the valves does not occur at this time point but in early Ventricular Ejection. Post-Ejection starts when the pulmonic and aortic valves are about to close, just before a backflow into the ventricles is generated. The forces of the backflow overcome the tension in the muscles and the ventricular volumes increase. This phase ends when the rapid relaxation starts and the tricuspid and the mitral valve are about to open, i.e. the muscle tension decreases which enables the returning action of the AV-pistons. This means that the flow over the tricuspid and mitral valves starts first during early Rapid Filling. Pre-Ejection, Ventricular Ejection and Post-Ejection in the regional and global state diagrams are according to these definitions different to what is commonly used when describing the isovolumic contraction and relaxation phases and the ejection. The phases Ventricular Ejection, Rapid Filling and Atrial Contraction were divided into subphases to improve the diagnostic power of the state diagram, as these subphases can provide further visual information concerning contractility of the heart muscles and flow and pressure conditions in the heart and its inlets and outlets.

### Clinical feasibility

The results of this study show that the state diagram has potential to be used for visualization of various cardiac dysfunctions. It also demonstrates the state diagram to be a more sensitive tool for detection of NSTEMI compared to other established echocardiographic variables such as E/E' ratio, LVEF and WMSI, since no significant differences were found in these variables. Moreover, it must be considered that the state diagram method had the ability to separate the groups, even though the control group demonstrated some clinical features. The novelty with this method is the visualization by presenting a clear overview of cardiac mechanics according to the DDP-principle. With this approach it is possible to create a state diagram, which allows for evaluation of parallel information by displaying the relationships between different phases in the cardiac cycle.

Though the state diagram method is based on TDI, its accuracy and reproducibility can be compared with the accuracy and the reproducibility established for TDI. TDI has been validated and a reasonably good reproducibility has been found [20,21]. However, the accuracy and reproducibility of the state diagram method has to be further addressed in future studies. Additionally, in comparison with methods quantifying cardiac function through single amplitude values of e.g. displacement, velocity and strain, the timing information used in the state diagram would most likely be less sensitive to noise, angular errors and filtering effects. The state diagram as a diagnostic tool could presumably be improved in future versions by extending it to contain information about, for example, stroke-length, auto-regulating functions of IVS [15], flows and pressures.

The pre- and postsystolic phases, called Pre-Ejection and Post-Ejection in this study, are important considerations when evaluating cardiac function and the prolongation of these time intervals are associated with systolic and diastolic ventricular dysfunction [22-24]. The results of the clinical study showed significantly prolonged Pre-Ejection and Post-Ejection for the NSTEMI subjects compared to the control subjects (p < 0.05), which has been quantitatively and fractionally presented in the state diagram in Figure 4. This is also clearly seen in the clinical examples in Figure 3, when comparing the Pre-Ejection and Post-Ejection for the healthy subject and the athlete with the diseased ones. One reason behind the short Post-Ejection phase noticed in the athlete in Figure 3b could be that an athlete has high persisting flow in the outflow vessels towards the end of Ventricular Ejection, which will reduce the pressure inside the ventricles, and thus the tension in the ventricular muscles, thereby shortening Post-Ejection. These dynamic features are also observed in the DDP and, in general shorter Pre-Ejection and Post-Ejection improve the dynamics in the heart, which could be an explanation for the improved ejection fraction seen in well-trained athletes [25].

Furthermore, the clinical examples of the ischemic and the dyssynchronic subjects indicate that the state diagram can detect and display regional heart muscle disturbances such as infarcted and ischemic movement patterns and dyskinetic functions of the muscle cells. Ventricular dyssynchrony disturbs the synchronous pumping and relaxation function of the heart leading to a reduced diastolic filling time and a post-systolic regional contraction [26], two factors that are easily detected in the state diagram. Coronary blood flow is impeded during systole which makes the duration of diastole an important determinant of myocardial perfusion. Adjustment of the diastolic time fraction has been proposed as a protective intervention to preserve coronary blood flow [27]. In left ventricular dyssynchrony, the regions with a dyssynchronic contracting myocardium pattern can be identified and the obtained information could potentially improve the selection of patients referred to cardiac resynchronization therapy. The state diagram acquired in the subject with dyssynchrony, Figure 3d, clearly visualizes how regional functions create impacts on all the phases in the global state diagram. A prolonged Post-Ejection, usually referred to as diastolic dysfunction in individuals with dyssynchroni disorders [28], can by visualization with the state diagram, be proven to originate from dyssynchrony occurring before this phase. Another reason for the prolonged Post-Ejection seen in the dyssyncronic subject, the ischemic subject and the NSTEMI-group could be low flow and high pressures in the outflow vessels towards the end of Ventricular Ejection, which would sustain the tension in the ventricular muscles and prolong the Post-Ejection. The significant difference between the groups found in late Atrial Contraction and early Rapid Filling indicates that muscular tension patterns differ between the groups. Indeed, previous studies have shown that degeneration of the elastic properties of the ventricles provokes a restrictive ventricular filling pattern characterized by a shortened early diastolic mitral flow deceleration time [29]. The corresponding time in the myocardial velocity curve is visualized in the state diagrams, giving an impression of the receiving performance of the ventricles. In cases with normal right atrial pressure, pulmonary artery systolic pressure (PASP) has been shown to correlate well with IVRT measured in the right ventricular free wall [30]. That IVRT is sensitive to changes in PASP should be considered when evaluating the right ventricular information in the state diagram.

### Limitations

Care must be taken when interpreting the results since the number of patients included in the study was small. The state diagrams for the patients in the clinical study and for the ischemic clinical example were generated using a 2D transducer. A 2D transducer renders the state diagrams dependent on recording conditions since the timing, based on velocities from different cardiac cycles, is displayed simultaneously. This is negligible in most cases since the beat-to-beat differences during an examination are small. In addition, the differences in heart rate between each NSTEMI patient and the matched control might have affected the results of the clinical study. However, these differences were not systematic and had most likely only a moderate influence on the results.

## Conclusion

The test results clearly indicate that the state diagram is feasible in the clinical setting and has potential to be an efficient tool for visualization of cardiac dysfunction and for use in the detection of NSTEMI. However, larger population studies are needed to further test the feasibility of state diagrams in this regard.

## Competing interests

Potential conflicts of interest are as follows: JJ is on the board in GrippingHeart AB and his Ph.D work is partly founded by GrippingHeart AB. SL partly owns GrippingHeart AB.

## Authors' contributions

ML, AB, JJ, SL and LÅB participated in contributions to conception, analysis and interpretation of data. ML, AB and JJ contributed with the programming work. ML and AB wrote the manuscript, critically reviewed by JJ and SL. RW was the supervisor of echo examinations and reviewed the manuscript for relevant intellectual content. All authors read and approved the final manuscript.
